# Chitosan Oligosaccharides Show Protective Effects in Coronary Heart Disease by Improving Antioxidant Capacity via the Increase in Intestinal Probiotics

**DOI:** 10.1155/2019/7658052

**Published:** 2019-03-10

**Authors:** Tiechao Jiang, Xiaohong Xing, Lirong Zhang, Zhen Liu, Jixue Zhao, Xin Liu

**Affiliations:** ^1^Department of Cardiovascular, China-Japan Union Hospital of Jilin University, Changchun 130033, China; ^2^Jilin Provincial Molecular Biology Research Center for Precision Medicine of Major Cardiovascular Disease, Changchun 130033, China; ^3^Department of Pathology, China-Japan Union Hospital of Jilin University, Changchun 130033, China; ^4^Department of Pediatrics, Liuhe District Hospital of Nanjing, Nanjing 211500, China; ^5^Department of Pediatrics Surgery, The First Hospital of Jilin University, Changchun 130021, China; ^6^Central Sterile Supply Department, China-Japan Union Hospital of Jilin University, Changchun 130033, China

## Abstract

We explored the effects of chitosan oligosaccharides (COS) on coronary heart disease (CHD) patients. The component of COS was measured by matrix-assisted laser desorption ionization time-of-flight mass spectrometry (MALDI-TOF MS). CHD patients were evenly assigned into the COS group (COG) and the placebo group (CG). The duration of treatment was 6 months and therapeutic results were explored by measuring left ventricular ejection fraction (LVEF) value, Lee scores, quality of life (QOL), blood urea nitrogen, and serum creatinine. The intestinal flora were determined by 16s rDNA sequencing. The circulating antioxidant levels and lipid profiles were compared between two groups. There were 7 different degrees of polymerization (DP4-10) in COS. Lee scores, QOL scores, and LVEF values in the COG group were higher than those in the CG group (*P* < 0.05). COS treatment improved blood urea nitrogen and serum creatinine when compared with controls (*P* < 0.05). Circulating antioxidant levels were higher in the COG group than in the CG group. COS consumption increased the serum levels of SOD and GSH and reduced the levels of ALT and AST (*P* < 0.05). Meanwhile, lipid profiles were improved in the COG group. COS consumption increased the abundance of *Faecalibacterium*, *Alistipes*, and *Escherichia* and decreased the abundance of *Bacteroides*, *Megasphaera*, *Roseburia*, *Prevotella*, and *Bifidobacterium* (*P* < 0.05). On the other hand, COS consumption increased the probiotic species *Lactobacillus*, *Lactococcus*, and *Phascolarctobacterium*. The increased species have been reported to be associated with antioxidant properties or lipid improvement. COS had similar effects with chitohexaose on the growth rate of these species. Therefore, COS ameliorate the symptoms of CHD patients by improving antioxidant capacities and lipid profiles via the increase of probiotics in the intestinal flora.

## 1. Introduction

Coronary heart disease (CHD) is mainly caused by circulating cholesterol accumulation on the artery walls, narrow arteries, and reduced blood flow to the heart [1]. CHD is a major cause of death worldwide and its prevalence is still increasing with population ageing [2]. CHD is difficultly diagnosed [3], and most medical treatment can cause side effects. Angiotensin-converting enzyme 2 (ACE2) is a regulator of the renin angiotensin system and has been widely used in the prevention of CHD development. Recent work showed that ACE2 treatment could increase the hazard of unwanted long-term cardiovascular outcomes in CHD patients [4]. Aspirin is also widely used for CHD therapy as anti-inflammatory pharmaceutical. Administration of aspirin may result in altered reproductive profiles and serum biochemistry [5]. Therefore, it is highly demanded to explore natural products with few side effects in the prevention of CHD risk and progression.

Chitosan is the second most abundant polysaccharide next to cellulose as a natural renewable resource. Chitosan oligosaccharides (COS) are effective antiatherosclerosis potential natural products [6] and have many valuable properties. COS exert obvious efficiency for preventing intestinal lipid absorption and improving liver lipid biosynthesis and accumulation [7] while lipid profile is an important risk factor of CHD [8]. Furthermore, COS have excellent biological properties presenting a promising prospect in antibacterial [9] and antioxidant biomaterials [10] with little cytotoxicity. Antioxidant therapy will be an effective way for CHD treatment since long-term hyperglycemia can result in the enhancement of oxidative stress [11]. Chitosan consumption can affect fecal microbiota and metabolites of humans [12], which may be associated with the changes of the intestinal flora. Intestinal flora disorder and disturbance also increase the CHD risk [13] and affect lipid metabolism [14] and antioxidant activities [15].

The above results suggest that COS are feasible and promising natural products for CHD patients. However, the effects of COS on CHD patients and the related molecular mechanisms remain unknown. Therefore, we explored the effects of COS on CHD patients and the changes of the intestinal flora. The improvement of the quality of life of CHD patients was compared with controls, and the levels of antioxidant and lipid profiles were measured.

## 2. Materials and Methods

### 2.1. Measurement of the Component of COS

Food-grade COS, chitohexaose hydrochloride (MW 1203.72), chitoheptaose hydrochloride (MW 1401.3), and chitooctaose hydrochloride (MW 1598.94) were purchased from Qingdao BZ Oligo Biotech Co. Ltd. (Qingdao, China). Thirty milligrams of COS or other oligosaccharides was dissolved in 1.0 mL ddH_2_O and transferred to a chromatography flask for the analysis of matrix-assisted laser desorption ionization time-of-flight mass spectrometry (MALDI-TOF MS). Voyager DESTR type MALDI-TOF mass spectrometer was purchased from Applied Biosystems (Carlsbad, CA, USA). The following operating parameters were used: nitrogen laser (wavelength 337 nm, pulse width 3 ns), reflection mode vacuum 2.08 × 10^−6^ Torr, ion source acceleration voltage 20 kV, extraction voltage 92.1%, and the ion delay 125 s. The mass spectrometry signal was accumulated 50 times in a single scan, and the positive ion model was determined.

### 2.2. Participants

All procedures were approved by the human research ethical committee of China-Japan Union Hospital of Jilin University (Changchun, China) (the clinical register no. ChiCTR1900020902 at http://www.chictr.org.cn/searchprojen.aspx). All patients agreed to sign the written consent form. CHD patients were diagnosed by using electrocardiogram, myocardial enzymology markers, coronary angiography, and clinical manifestations.

### 2.3. Inclusion Criteria

The patients had clinical manifestations of typical pain, which mostly occurred in the early morning. Sudden and severe stern poststernal or precordial compression pain could be found but the cause was not obvious. Taking a rest or taking nitroglycerin tablets could not alleviate the symptoms. The patients were often upset, sweating, fearful, experienced chest tightness, or had a sense of death. Typical electrocardiogram showed that ST-segment elevation was arch-back-up with wide and deep Q wave (pathological Q wave) and T wave inversion. The levels of serum myocardial necrosis markers, myoglobin and troponin I (cTnI), or myocardial markers, such as cardiac troponin T (cTnT) and creatine kinase isoenzyme CK-MB, were significantly elevated.

### 2.4. Exclusion Criteria

The patients with the following condition were excluded: (1) gastrointestinal diseases, such as gastritis and gastric ulcer, and diarrhea in the past 4 weeks or a history of gastrointestinal surgery; (2) combined symptoms of heart failure; (3) cardiogenic shock; (4) previous coronary revascularization procedures (e.g., thrombolysis and PCI); (5) combined symptoms with autoimmune diseases; (6) other endocrine diseases such as thyroid disorder; (7) serious damage to organs such as the liver and kidney; (8) oral and intravenous antibiotic administration for nearly 1 week and adjustment of intestinal flora preparation and gastric mucosal protective agent for nearly 1 week; (9) hypertension, obesity, diabetes, and dyslipidemia; and (10) pregnant.

### 2.5. Patient Grouping

From March 4, 2016, to April 28, 2017, a total of 528 CHD patients were screened. The first primary endpoint was mortality, stroke, and myocardial infarction and the endpoint was determined according to a one-month observation after randomization. Finally, a sample size with 120 subjects was determined. COS have been sold widely in China as healthy products. The dosage of COS (1-2 g/day) was provided according to product instructions. COS mixtures administered orally at doses between 50 and 1,000 mg/kg b.w. will not produce any significant change in the autonomic or behavioral responses in animal models [16]. To maintain the safety of COS, the lowest dose of COS (2 g daily) was used. All patients were selected and evenly assigned into the COG (received 2 g COS daily) and CG (placebo) groups. The therapeutic duration was half a year.

### 2.6. Specimen Collection

Fresh stools were collected on the first morning after admission, and stool samples from all subjects were collected in closed fecal storage boxes within two-hour defecation (subjects took hospital diet and normal control diet). The samples were immediately stored in a -80°C refrigerator.

### 2.7. Extraction of Total DNA

Two-gram feces was placed in a 2.0 mL Safe-Lock tube, and glass beads were added and one-milliliter PBS (50 mM, pH 7.0) and vortexed evenly. The mixture was water bathed at 95°C for 10 min, and 20 *μ*L proteinase K was added and incubated at 55°C for 10 min. After centrifugation at 12000 rpm for 15 min, the supernatant was placed in a two-milliliter test tube. Genomic DNA was extracted and purified by using the kit from Promega (Madison, WI, USA). The quality of genomic DNA was determined by using Thermo NanoDrop 2000 Ultraviolet Micro Spectrophotometer (Thermo Fisher Scientific Inc., Waltham, MA, USA) and 1% agarose gel electrophoresis.

### 2.8. 16S rDNA Sequencing

The 16S rDNA amplification selection region is V3-V4 region, and a universal primer is used. The specific universal primers (forward primer: 5′-ACTCCTACGGGRSGCAGCAG-3′; reverse primer: 5′-GGACTACVVGGGTATCTAATC-3′) were used for 16S rDNA sequencing. The primers were completed by adding the index sequence and the linker sequence suitable for PE250 sequencing at the 5′ end of the primer. Using the diluted genomic DNA as a template, PCR was performed with KAPA HiFi HotStart ReadyMix PCR kit high-fidelity enzyme (Kapa Biosystems Inc., Boston, MA, USA). The PCR product was detected by 2% agarose gel electrophoresis, and the PCR product was recovered by gelatinization using an AxyPrep DNA Gel Recovery Kit (Axygen Scientific Co., CA, USA). After recovery, library quality checks were performed using a Thermo NanoDrop 2000 UV spectrophotometer and 2% agarose gel electrophoresis. PCR products were sequenced by using illumina HiSeq PE250 (Illumina, San Diego, CA, USA).

### 2.9. The Effects of COS on the Growth of Intestinal Flora

The strains *Bacteroides thetaiotaomicron* (CGMCC 1.5132, broken meat medium); *Escherichia coli* (CGMCC 1.12883, LB medium); *Megasphaera elsdenii* (CGMCC 1.2720, CGMCC medium 0288); and *Bifidobacterium bifidum* (CGMCC 1.5091, CGMCC medium 0244) were purchased from the China General Microbiological Culture Collection Center, Institute of Microbiology, Chinese Academy of Sciences (Beijing, China). The strains *Faecalibacterium prausnitzii* (ATCC 27768, ATCC medium: 2107 modified reinforced clostridial); *Alistipes shahii* (ATCC BAA-1179, ATCC medium 1490: modified chopped meat medium); and *Prevotella bivia* (ATCC 29303, ATCC medium 2107) were purchased from the American Type Culture Collection (ATCC) (Manassas, VA, USA). The strain *Roseburia intestinalis* (DSM 14610, medium 143) was purchased from Leibniz-Institut DSMZ-Deutsche Sammlung von Mikroorganismen und Zellkulturen GmbH (Inhoffenstraße, Braunschweig, Germany). *Escherichia coli* was cultured at a 200 rpm shaker at 37°C while other strains were cultured with anaerobic gas mixture, 80% N_2_, 10% CO_2_, and 10% H_2_ at 37°C with corresponding media and 10 *μ*g/mL chitohexaose hydrochloride, chitoheptaose hydrochloride, chitooctaose hydrochloride, and mixed 80 *μ*g/mL chitosan oligosaccharide. The group without chitosan oligosaccharides was used as a control. The growth rate of all strains was measured by using a Real-Time Cell Analyzer (xCELLigence™, Roche Inc., Indianapolis, IN, USA) within 24 hours.

### 2.10. Lipid Profile Analysis

Serum triglycerides (TG), total cholesterol (TC), low-density lipoprotein cholesterol (LDL-C), and high-density lipoprotein cholesterol (HDL-C) were examined by using an automatic biochemical analyzer (Dimension, Schererville, IN, USA).

### 2.11. Analyses of Circulating Oxidative Levels

2 mL blood was taken from individual patient. Circulating oxidative levels were examined by measuring the levels of superoxide dismutase (SOD), glutathione (GSH), alanine aminotransferase (ALT), and aspartate aminotransferase (AST). ELISA kits were purchased from Beyotime Institute of Biotechnology (Beijing, China).

### 2.12. Measurement of Therapeutic Effects

The normal therapy of CHD included oxygen inhalation, angiotensin-converting enzyme inhibitors (ACE-in), and implantable cardioverter defibrillator (ICD). Ejection fraction (EF) value and scores of quality of life (QOL) were evaluated by using the Minnesota Living with Heart Failure Questionnaire (MLHFQ) [17].

Left ventricular ejection fraction (LVEF) was detected by using radionuclide ventriculography with patients in the supine position [18]. Serum was separated from the blood sample via centrifugation at 4000 × *g* for 10 min. Blood urea nitrogen (BUN) was analyzed by using the above automatic biochemistry analyzer via a BUN kit (Beckman Coulter Inc., Brea, CA, USA). Serum creatinine was measured by a creatinine kit (Biosino Bio-Technology, Beijing, China).

### 2.13. Statistical Analysis

All data were presented as mean values ± S.D. and analyzed by using SPSS 20.0 statistical package. The *t*-test was used for the comparison of mean values between the two groups and count number was analyzed using the *χ*^2^ test. *P* < 0.05 was considered statistically significant.

## 3. Results

### 3.1. The Main Components of COS

**Figure 1:**
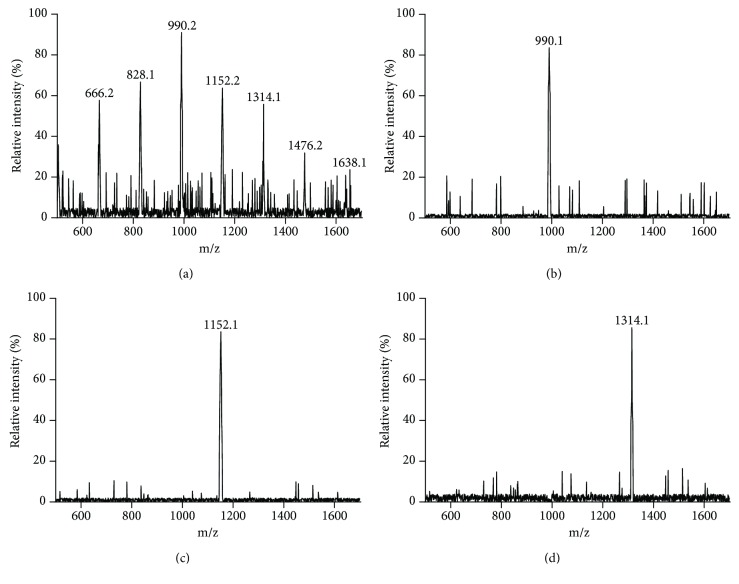
Matrix-assisted laser desorption ionization time-of-flight mass spectrometry (MALDI-TOF MS) analysis of the main components of chitosan oligosaccharides (COS). (a) The main components of food-grade COS with different degrees of polymerization DP4-10. (b) Chitohexaose with molecular weight [M+H]^+^ = 990.1 Da. (c) Chitoheptaose with molecular weight [M+H]^+^ = 1152.1 Da. (d) Chitooctaose with molecular weight [M+H]^+^ = 1314.2 Da.

There were seven main kinds of chitosan oligosaccharides in food-grade COS from DP4 (666.2 Da) to DP10 (1638.1 Da) (Figure 1(a)). The molecular weight of chitohexaose (990.1 Da, Figure 1(b)), chitoheptaose (1152.1 Da, Figure 1(c)), and chitooctaose (1314.1 Da, Figure 1(d)) were also accordant with theoretical values in a positive mode.

### 3.2. Clinical Characteristics

After the 6-month therapy, 4 and 6 patients left the COG and CG groups for other medical treatment, respectively. There was no significantly statistical differences for clinical characteristics of CHD patients between the COG and CG groups, including sex ratio, body mass index (BMI), age, diastolic blood pressure (DBP), and systolic blood pressure (SBP) (Table 1, *P* > 0.05). The cases for taking ACE-In, ARBS, beta-blockers, and diuretics were also comparable between the two groups (*P* > 0.05, Table 1).

### 3.3. Therapeutic Results

There was no significant difference (*P* > 0.05) in the mean values of BUN before COS therapy (*P* < 0.05, Table 2). After therapy, the values of mean BUN and serum creatinine were significantly reduced when compared with the placebo group (*P* < 0.05, Table 2). Before COS treatment, the statistical difference for Lee scores was insignificant between the COG and CG groups (Table 2, *P* > 0.05). After COS consumption, COS reduced more Lee scores than CG (Table 2, *P* < 0.05). Before COS consumption, the statistical difference for the QOL scores was insignificant between the COG and CG groups (Table 2, *P* > 0.05). After 6-month COS consumption, COS increased more QOL scores than CG (Table 2, *P* < 0.05). Before COS consumption, the statistical difference for LVEF volume was insignificant between the COG and CG groups (Table 2, *P* > 0.05). After 6-month COS consumption, the values of LVEF were improved in the COG group higher than in the CG group (Table 2, *P* < 0.05).

### 3.4. The Effects of COS Consumption on Intestinal Flora of CHD Patients

**Figure 2:**
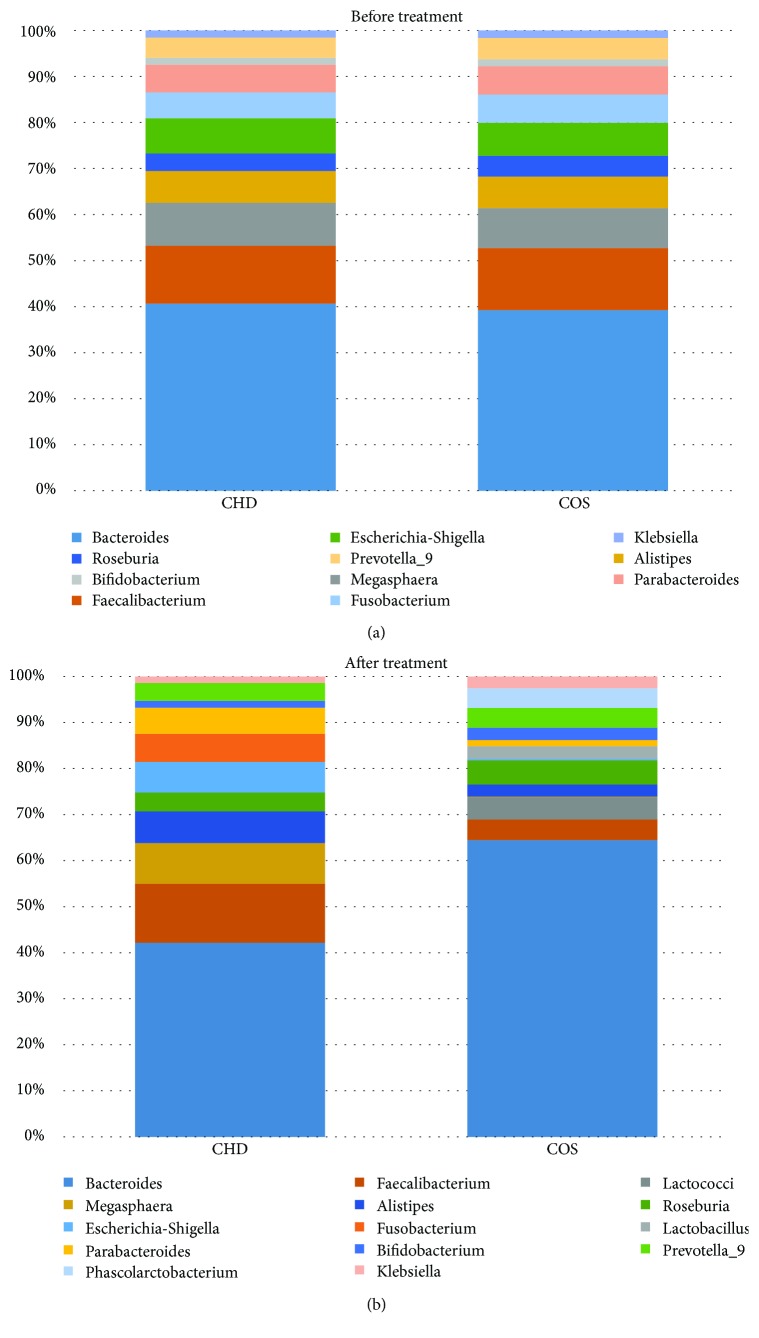
The effects of COS on intestinal flora. (a) The abundance of intestinal flora before COS treatment. (b) The abundance of intestinal flora after 6-month treatment.

The statistical difference for the abundance of intestinal flora was insignificant between the two groups before therapy (Figure 2(a)*P* > 0.05). After 6-month therapy, the abundance of *Faecalibacterium*, *Alistipes*, and *Escherichia* was reduced, while the abundance of *Bacteroides*, *Megasphaera*, *Roseburia*, *Prevotella*, and *Bifidobacterium* was increased when compared with the CG group (Figure 2(b), *P* < 0.05). On the other hand, COS consumption increased the probiotic species *Lactobacillus*, *Lactococcus*, and *Phascolarctobacterium*. The results suggest that COS consumption can inhibit the abundance of harmful bacteria and increase the abundance or species of probiotics in CHD patients.

### 3.5. The Effects of COS on the Growth Rate of Intestinal Flora

**Figure 3:**
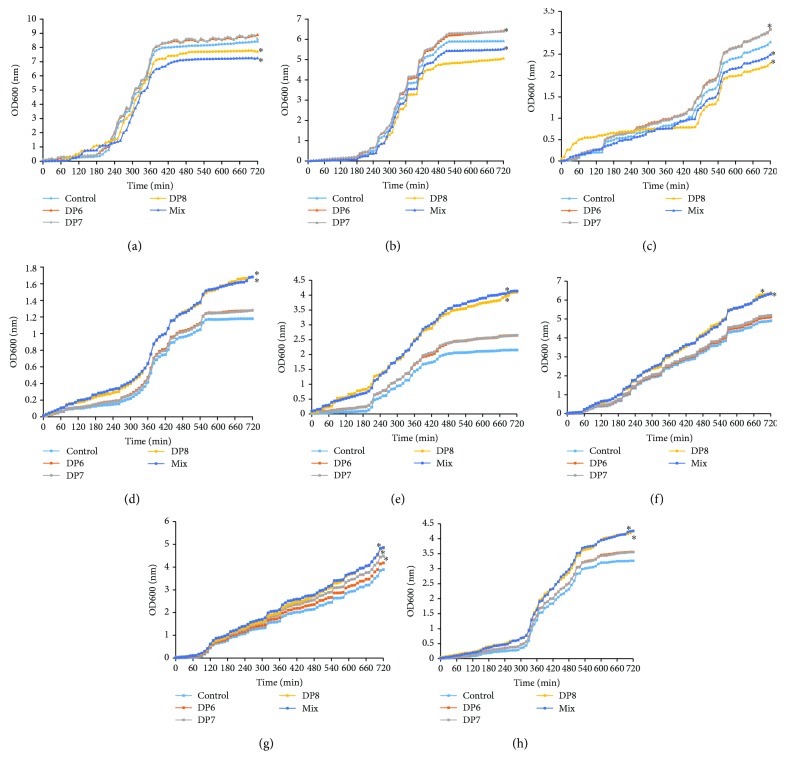
Real-time analysis of the effects of COS on the growth of intestinal flora. (a) The effects of COS on the growth of *Escherichia coli*. (b) The effects of COS on the growth of *Megasphaera elsdenii*. (c) The effects of COS on the growth of *Faecalibacterium prausnitzii*. (d) The effects of COS on the growth of *Alistipes shahii*. (e) The effects of COS on the growth of *Prevotella bivia*. (f) The effects of COS on the growth of *Roseburia intestinalis*. (g) The effects of COS on the growth of *Bacteroides thetaiotaomicron*. (h) The effects of COS on the growth of *Bifidobacterium bifidum*. Mix: food-grade COS, DP4-10 chitosan oligosaccharides; DP6: chitohexaose hydrochloride (MW 1203.72); DP7: chitoheptaose hydrochloride (MW1401.3); and DP8: chitooctaose hydrochloride (MW1598.94). ^∗^*P* < 0.05 vs. the control group without COS.

In vitro test showed that COS mixture and chitooctaose (DP8) treatment inhibited the growth rate of *Escherichia coli* (Figure 3(a)), *Megasphaera elsdenii* (Figure 3(b)), and *Faecalibacterium prausnitzii* (Figure 3(c)) and promoted the growth of *Alistipes shahii* (Figure 3(d)), *Prevotella bivia* (Figure 3(e)), *Roseburia intestinalis* (Figure 3(f)), *Bacteroides thetaiotaomicron* (Figure 3(g)), and *Bifidobacterium bifidum* (Figure 3(h)). Meanwhile, chitooctaose had similar results with mixed COS. Comparatively, chitohexaose and chitoheptaose had no effects on these bacteria. The results suggest that COS affect intestinal flora via chitooctaose.

### 3.6. COS Consumption Improved Lipid Profiles of CHD Patients

Before COS consumption, the statistical difference was insignificant between the two groups (*P* > 0.05, Table 3). After 6-month therapy, the serum levels of TG, TC, and LDL-c were reduced while HDL-c was increased when compared with the control group (*P* < 0.05, Table 3). The results suggest that COS consumption can improve lipid profiles of CHD patients.

### 3.7. COS Consumption Increased Antioxidant Properties of CHD Patients

The statistical difference for the biomarkers of antioxidant and oxidative stress was insignificant between the two groups before therapy (Table 4, *P* > 0.05). After 6-month therapy, the circulating levels of SOD and GSH were increased while the levels of ALT and AST were reduced in the COG group when compared with the CG group (*P* < 0.05, Table 4). The results suggest that COS consumption can increase antioxidant properties of CHD patients.

## 4. Discussion

Chitosan has been widely used for CHD therapy as biomaterials of the drug delivery system [19, 20] and coronary artery bypass graft [21]. However, the direct effects of chitosan on CHD have seldom been reported. This study showed that COS consumption increased the values of LVEF in the COG group higher than in the CG group (*P* < 0.05). Medicine combined with COS effectively ameliorated CHD patients with lower LVEF. Lee scores and QOL scores were also increased in the COG group (*P* < 0.05, Table 2). Meanwhile, lipid profiles (Table 3) and antioxidant properties (Table 4) were also improved in the COG group better than the CG group (*P* < 0.05). The results suggest that COS are effective to ameliorate symptoms of CHD patients by improving biochemical indices and the living ability of CHD patients.

LVEF is an important predicator for heart failure hospitalization and mortality in ambulatory adults with CHD [22]. CHD patients with low-level LVEF had a poor prognosis [23]. The present findings demonstrated that COS consumption improved LVEF values significantly when compared with controls (Table 2, *P* < 0.05). COS have been reported to have the clinical effects of pain relief [24], which will be beneficial for CHD patients with chest pain or chronic back pain [25, 26]. Chitosan microspheres were used for chronotherapy of chronic stable angina in an animal model [27] while angina is the common symptom of CHD patients [28, 29]. Fatigue is a prevalent and disabling symptom associated with CHD [30] while COS have been proved to delay fatigue in animal models [31]. All these results suggest that COS may have direct or indirect effect on improving CHD symptoms.

On the other hand, intestinal flora total load was found to be associated with CHD risk in obese patients [13]. Intestinal flora can produce short-chain fatty acids (SCFA) [32, 33] and bile acids [34, 35] involved in various metabolic pathways, endotoxin secretion and circulation, dopamine, the fasting-induced adipose factor (FIAF) [36], and adenosine monophosphate-activated protein kinase (AMPK) [37], which affect CHD risk factors such as hypertension [38], obesity [39], diabetes [40], and dyslipidemia [41]. Adjusting the structure and function of the flora via probiotics, antibiotics or diet can provide ideal methods for the prevention of CHD. Therefore, the effects of COS on intestinal flora were explored in CHD patients.

**Figure 4:**
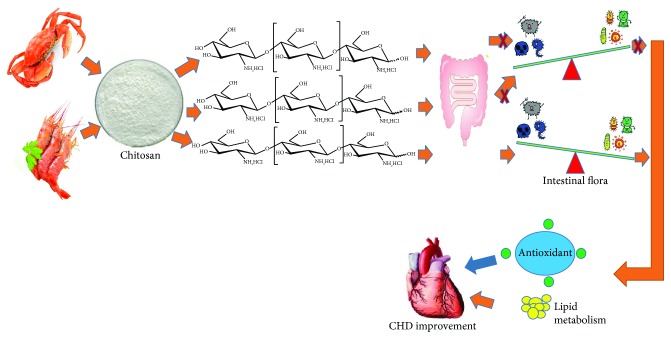
COS show health-promoting properties for coronary heart disease by affecting intestinal flora via chitooctaose. COS increase the levels of probiotics, which exert antioxidant properties and improve lipid profiles. All the function will be beneficial in the prevention of CHD.

After COS consumption, the abundance of *Faecalibacterium*, *Alistipes*, and *Escherichia* was reduced, while the abundance of *Bacteroides*, *Megasphaera*, *Roseburia*, *Prevotella*, and *Bifidobacterium* was increased (Figure 2(b)). Furthermore, COS consumption increased the probiotic species *Lactobacillus*, *Lactococcus*, and *Phascolarctobacterium*. *Faecalibacterium* was found to be linked with type 2 diabetes mellitus or other risk factors of heart disease [42]. Conversely, *Faecalibacterium* numbers were also found to be decreased in the patients with chronic heart disease [43]. Cardiac abnormalities were reported to be caused by bacterial myocarditis resulting from *E. coli* infection [44, 45]. A high-fiber diet and supplementation with the SCFA can increase the number of *Bacteroides acidifaciens* and prevent cardiovascular disease [46]. *Roseburia* reduced microbially derived, proinflammatory secondary bile acids and LDL-c, which are associated with heart disease [47]. *Bifidobacterium* exerted beneficial effects on the serum cholesterol metabolism by reducing the levels of TC and LDL-c in the patients with dyslipidemia [48]. *Bifidobacterium* had strong antioxidant properties by scavenging 2,2-diphenyl-1-picryl-hydrazyl (DPPH) radical, superoxide anion, and hydroxyl radical [49]. However, *Prevotella* was reported to be a potential risk bacterium of heart disease [50]. All the growth of these species could be affected by COS while COS had similar results with chitooctaose but not chitohexaose and chitoheptaose (Figure 3). Thus, the COS from crab and shrimp may ameliorate CHD by affecting intestinal flora, which improve lipid profiles and antioxidant activities of CHD patients (Figure 4).

More importantly, the increased probiotic species *Lactobacillus* and *Lactococcus* show health-promoting activities for CHD. *Lactobacillus* showed strong antioxidant function by upregulating the expression of glutathione reductase, glutathione S-transferase, glutamate-cysteine ligase catalytic subunit, and NAD(P)H quinone oxidoreductase 1 [51]. *Lactococcus acidophilus* was found to prevent the progression of arteriosclerosis and coronary heart disease by affecting lipid profiles and increasing the antioxidant abilities of hyperlipidemia animal model [52].

The lipid metabolism may be correlated with not only intestinal flora but also intestinal enzymes. Previous work showed that intestinal disaccharidases (such as sucrase and maltase) were significantly decreased animals being fed with COS [53]. COS were proved to have antihyperglycemia ability for inhibiting carbohydrate hydrolysis enzymes, such as sucrase and glucoamylase [54]. Based on these findings, further clinical trials are highly demanded to prove the mechanism.

There were some limitations to the present work. The effects of chitosan oligosaccharides DP4-5 and DP9-10 on intestinal flora were not investigated although the mixed COS had similar results with chitooctaose for the growth rate of intestinal flora. The effects of COS on the increased probiotic species *Lactobacillus*, *Lactococcus*, and *Phascolarctobacterium* were not measured and reason remained unknown. Considering the short time of the present study, the small sample size, and other influencing factors, further work is highly demanded to confirm the present results.

## 5. Conclusions

COS combined with conventional treatment improved the LVEF values, QOL scores, and Lee scores. COS consumption increased the types and numbers of probiotic species of intestinal flora, which may improve lipid profiles and antioxidant properties of CHD patients. COS had similar effects with chitohexaose on the growth rate of these species. Therefore, COS improve the symptoms of CHD patients by improving antioxidant capacities via the increase of probiotics in intestinal flora.

## Figures and Tables

**Table 1:** Clinical characteristics between COS and placebo groups.

Parameters	COS	Placebo	*χ* ^2^ and *t* value	*P* value
Gender (male/female)	30/26	31/23	0.164	0.686
Age (yr)	39.29 ± 13.36	41.23 ± 12.98	-1.307	0.189
SBP (mm Hg)	125.21 ± 11.62	128.54 ± 12.76	-1.685	0.087
DBP (mm Hg)	87.23 ± 7.16	88.53 ± 7.38	-1.290	0.157
BMI	24.93 ± 2.94	24.52 ± 2.68	-1.564	0.198
Cr (*μ*mol/L)	85.34 ± 13.58	87.24 ± 14.56	-1.344	0.156
HbA1C (%)	8.47 ± 0.93	8.75 ± 0.96	-0.664	0.256
ACE-In	7	8	2.47	0.48
ARBS	3	6		
Beta-blockers	5	6		
Diuretics	8	4		

Chi-square test and *t*-test were used to compare the significant difference between the two groups. BMI: body mass index; ACE-In: angiotensin-converting enzyme; ARBS: angiotensin receptor blockers. All data were presented as mean value ± S.D. There were significantly statistical differences between two groups if *P* < 0.05.

**Table 2:** The therapeutic results of COS.

		Before treatment	After treatment	*t* values	*P* values
Blood urea nitrogen (mg/dL)	COS	19.13 ± 6.85	15.33 ± 6.24	6.42	0.02^b^
	Placebo	18.73 ± 6.54	17.25 ± 5.98	1.16	0.23
	*t* values	0.39	3.21		
	*P* values	0.54	0.02^a^		
Serum creatinine (mg/dL)	COS	1.41 ± 0.32	1.04 ± 0.27	8.65	0.01^b^
	Placebo	1.35 ± 0.27	1.29 ± 0.22	0.35	0.24
	*t* values	0.25	4.37		
	*P* values	0.66	0.02^a^		
Lee scores	COS	4.52 ± 1.87	2.18 ± 0.43	5.38	0.01^b^
	Placebo	4.27 ± 1.79	4.19 ± 0.78	0.25	0.31
	*t* values	0.26	4.12		
	*P* values	0.68	0.02^a^		
Quality-of-life (QOL) scores	COS	43.61 ± 3.38	21.73 ± 4.12	13.40	0.01^b^
	Placebo	42.50 ± 3.25	39.39 ± 4.36	0.45	0.29
	*t* values	0.36	2.13		
	*P* values	0.75	0.02^a^		
LVEF	COS	29.06 ± 9.34	36.82 ± 10.43	3.03	0.02^b^
	Placebo	28.74 ± 8.15	30.73 ± 10.21	1.10	0.08
	*t* values	0.92	2.17		
	*P* values	0.36	0.03^a^		

Note: LVEF: left ventricular ejection fraction. *n* = 60 for each group. ^a^*P* < 0.05 vs. the placebo group and ^b^*P* < 0.05 vs. before treatment. There were significantly statistical differences between the two groups if *P* < 0.05.

**Table 3:** Lipid profiles between two groups.

		COS	Placebo	*t* values	*P* values
Before therapy	TC (mmol/L)	5.42 ± 0.63	5.70 ± 0.81	-0.621	0.284
	TG (mmol/L)	2.34 ± 0.81	2.17 ± 0.92	-2.108	0.129
	LDL-C (mmol/L)	2.11 ± 0.62	2.31 ± 0.81	-1.834	0.167
	HDL-C (mmol/L)	1.83 ± 0.42	1.65 ± 0.38	-2.609	0.094
After therapy	TC (mmol/L)	4.89 ± 0.87	5.81 ± 0.72	-1.982	0.013
	TG (mmol/L)	2.01 ± 0.65	2.24 ± 0.83	-2.696	0.035
	LDL-C (mmol/L)	1.81 ± 0.54	2.40 ± 0.75	-1.992	0.031
	HDL-C (mmol/L)	2.13 ± 0.40	1.71 ± 0.46	-2.852	0.009

Note: there were significant statistical differences between two groups if *P* < 0.05.

**Table 4:** Antioxidant levels between two groups.

		COS	Placebo	*t* values	*P* values
Before therapy	SOD (U/mL)	12.25 ± 4.06	11.30 ± 4.21	0.79	0.46
	GSH (U/mL)	9.20 ± 2.99	9.35 ± 2.73	0.12	0.81
	ALT (U/mL)	68.79 ± 9.03	65.52 ± 8.76	0.35	0.47
	AST (U/mL)	198.21 ± 27.21	190.36 ± 20.38	0.83	0.12
After therapy	SOD (U/mL)	21.34 ± 3.78	13.49 ± 4.71	8.77	0.01
	GSH (ng/L)	15.26 ± 3.12	10.59 ± 2.01	6.25	0.01
	ALT (U/L)	61.03 ± 12.27	66.49 ± 8.24	4.31	0.02
	AST (U/L)	147.43 ± 21.73	188.52 ± 23.62	7.04	0.01

Note: there were significant statistical differences between two groups if *P* < 0.05.

## Data Availability

The data used to support the findings of this study are available from the corresponding author upon request.
